# Biological Functions of Strigolactones and Their Crosstalk With Other Phytohormones

**DOI:** 10.3389/fpls.2022.821563

**Published:** 2022-02-24

**Authors:** Fenghui Wu, Yinping Gao, Wenjing Yang, Na Sui, Jianping Zhu

**Affiliations:** Shandong Provincial Key Laboratory of Plant Stress, College of Life Sciences, Shandong Normal University, Jinan, China

**Keywords:** strigolactones, development, phytohormones, crosstalk, signalling pathway

## Abstract

Phytohormones are small chemicals critical for plant development and adaptation to a changing environment. Strigolactones (SLs), carotenoid-derived small signalling molecules and a class of phytohormones, regulate multiple developmental processes and respond to diverse environmental signals. SLs also coordinate adjustments in the balance of resource distribution by strategic modification of the plant development, allowing plants to adapt to nutrient deficiency. Instead of operating independently, SL interplays with abscisic acid, cytokinin, auxin, ethylene, and some other plant phytohormones, forming elaborate signalling networks. Hormone signalling crosstalk in plant development and environmental response may occur in a fully concerted manner or as a cascade of sequential events. In many cases, the exact underlying mechanism is unclear because of the different effects of phytohormones and the varying backgrounds of their actions. In this review, we systematically summarise the synthesis, signal transduction, and biological functions of SLs and further highlight the significance of crosstalk between SLs and other phytohormones during plant development and resistance to ever-changing environments.

## Introduction

Plants are frequently exposed to diverse unfavourable environmental conditions that lead to abiotic stresses and reduce productivity. Phytohormones are crucial for regulating various physiological processes of plants and assisting them to communicate with the external environment (Ciura and Kruk, 2018; Xin et al., 2019; Li et al., 2020). Strigolactones (SLs) were discovered when analysing the ability of a signalling substance secreted by cotton roots to stimulate the seed germination of parasitic weeds (Cook et al., 1966). Approximately 25 types of naturally occurring SLs have been discovered in different plant species, and based on their chemical structures, they are classified into two groups, namely, canonical and non-canonical SLs (Wang and Bouwmeester, 2018). Canonical SLs consist of a butenolide ring (D ring) connected by an enol ether bridge to a tricyclic lactone (ABC rings) (Butler, 1995). In non-canonical SLs, the ABC ring is replaced with an irregular ring structure (Yoneyama et al., 2018). Different forms of SL molecules may exhibit different biological activities (Umehara et al., 2015; Xie et al., 2020). The complex structure and stereochemistry of natural SLs limit their chemical synthesis. GR24, a synthetic SL analogue widely used in SL studies, is a racemic mixture of two 5-deoxystrigol (5DS)-configured enantiomers, namely, GR24^5DS^ and GR24^ent–5DS^ (Yao et al., 2021).

Nitrogen (N) and phosphorus (P) are essential macronutrients for plants. As signalling mediators, SLs regulate the coordinated development of roots and shoots, particularly under N- and P-deficient conditions (Sun et al., 2014; Ito et al., 2015; Xi et al., 2015). Accordingly, SLs regulate above- and belowground plant morphogenesis, including shoot branching, leaf senescence, reproductive development, adventitious root (AR) formation, and root hair (RH) density (Kretzschmar et al., 2012; Yamada et al., 2014; Sun J. et al., 2015; Tan et al., 2019; Mitra et al., 2021). Moreover, a continuously increasing number of studies have suggested that SLs confer tolerance to different suboptimal growth conditions, especially drought and salinity (Saeed et al., 2017; Zhang X. et al., 2020). All these functions require coordinated changes at the molecular level in a complex plant growth network, necessitating the communication and cooperation of two or more hormone signals. The crosstalk between SL and other signalling pathways regulated by phytohormones, such as auxin, cytokinin (CK), ethylene (ET), and abscisic acid (ABA), has attracted extensive attention. This review verifies the latest information concerning the biological functions of SLs and further broadens and clarifies SL-associated hormonal networks in plant development and responses to several environmental challenges.

### Strigolactones: Biosynthesis and Signalling Transduction

**FIGURE 1:**
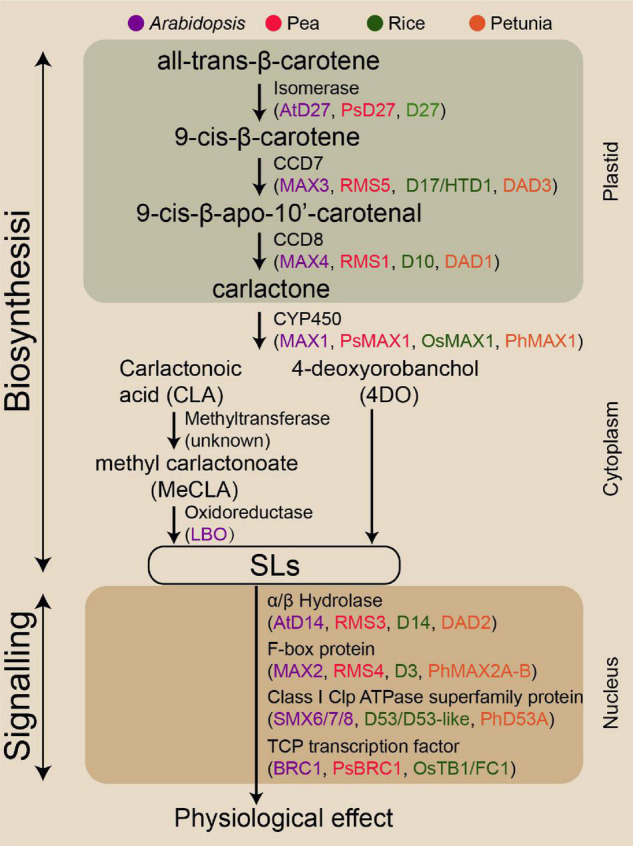
Genes encoding the enzymes involved in SL biosynthesis and signalling pathway identified in the four model species.

Given the benefits of SLs in plant biology, SLs exhibit the potential to improve crop genotypes with enhanced abiotic stress resilience and crop productivity. Understanding and exploiting SL biosynthesis are critical for effectively translating this potential into the modern agriculture industry. Although the SL biosynthesis pathway has not been fully elucidated, most enzymes involved in this pathway have been identified (Figure 1). SLs are plant secondary metabolites synthesised from carotenoids, which are converted to the SL precursor carlactone (CL) by the carotenoid isomerase DWARF27 (D27) and two carotenoid cleavage dioxygenase genes, namely, CCD7 and CCD8 (Lin et al., 2009; Alder et al., 2012). In *Arabidopsis*, MORE AXILLARY GROWTH1 (MAX1) encodes a cytochrome P450 monooxygenase (CYP711A1) that catalyses the conversion of CL to produce carlactonoic acid (CLA), which is then methylated to methyl carlactonoate (MeCLA) by an unknown methyltransferase (Abe et al., 2014; Seto et al., 2014). Lateral branching oxidoreductase is responsible for the oxidation of MeCLA into the SL-like compound (Brewer et al., 2016). In contrast to *Arabidopsis*, the rice MAX1 homologue Os900 (CYP711A2) converts CL into 4-deoxyorobanchol (4DO), and finally another homologue, Os1400 (CYP711A3), further catalyses the 4DO to form orobanchol (Zhang et al., 2014).

Strigolactone signalling transduction mechanisms are similar to those of other plant phytohormones. These mechanisms involve hormone-activated targetting of transcriptional regulators for degradation, likely involving α/β-fold hydrolases DWARF14 (D14 in rice) and F-box component (MAX2 in *Arabidopsis*) (Hamiaux et al., 2012; Nakamura et al., 2013). The D14 has a conserved catalytic triad (Ser-His-Asp) with hydrolase activity for recognising and deactivating SL (Yao et al., 2016). KARRIKIN INSENSITIVE2 (KAI2) is structurally closely related to D14 that perceives smoke-derived karrikin (KAR) and non-naturally derived SL enantiomers such as GR24^ent–5DS^ (Waters et al., 2012). SL molecules bind to D14, resulting in the conformational change of D14, thereby facilitating D14 interaction with F-box proteins MAX2 (Zhao et al., 2015). This complex triggers the ubiquitination of transcriptional repressor D53 (homologous SMXL6, SMXL7, or SMXL8 in *Arabidopsis*), resulting in the 26S proteasomal degradation of this repressor and thus the transcription of SL responsive genes (Jiang et al., 2013; Wang et al., 2015; Yao et al., 2018). D53 is a key target in controlling axillary bud outgrowth in rice (Jiang et al., 2013; Fang et al., 2020). The SPL family transcription factor Ideal Plant Architecture1 in rice and D53 together mediates the transcriptional activation of genes in the SL regulatory process (Song et al., 2017). Numerous studies using various SL biosynthesis and signalling lines have demonstrated that SLs can positively modulate RH elongation, primary root (PR) growth, and secondary shoot growth, but repress AR development and axillary bud outgrowth (Agusti et al., 2011; Koltai, 2011; Rasmussen et al., 2012).

## Biological Functions of Strigolactones

### Effect of Strigolactones on Shoot Architecture and Root Development

**FIGURE 2:**
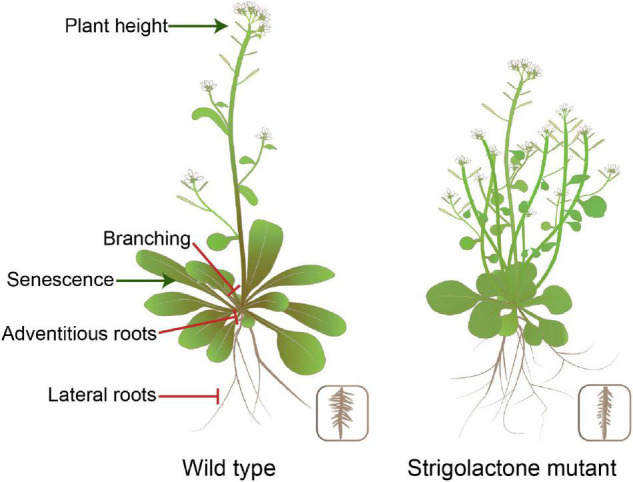
Impact of SL production levels on plant morphology in *Arabidopsis*. SLs are involved in various developmental processes, including plant height, shoot branching, and root system architecture.

Strigolactones are a class of phytohormones shaping the overall plant structure. For example, they control shoot branching, secondary growth, and root morphology. Shoot branching patterns result from the regulation of axillary bud growth. Many endogenous and external signals determine the growth or dormancy of each axillary bud (Qiu et al., 2019; Xie et al., 2020). Apical dominance is a phenomenon in which bud outgrowth is inhibited by the apex of the main shoot. Part of the inhibitory effect of apical dominance on bud outgrowth is due to the production of auxin by the apical young leaves (Domagalska and Leyser, 2011). However, auxin does not enter the buds and acts interdependently, partly by inducing strigolactone synthesis (Rameau et al., 2015; Wang H. W. et al., 2018; Barbier et al., 2019). Both SL biosynthesis and signalling-deficient mutants are semi-dwarf and exhibit increased branching, which gives the mutants a bushy appearance in *Arabidopsis* (Figure 2). SL mediates axillary bud outgrowth. This process involves SL-induced upregulation of the TCP transcription factor BRANCHED1 (BRC1) that suppresses bud activity (Braun et al., 2012; Dun et al., 2013). In addition, auxin flow out of axillary buds is contributing to bud outgrowth, and SL inhibition of the auxin efflux carrier PIN1 localisation to the plasma membrane and/or the effect of SL on auxin feedback on PIN1 internalisation reduce auxin efflux from lateral buds, thus enhancing competition among buds in the stem (Crawford et al., 2010; Waldie et al., 2014; Brewer et al., 2015; Zhang J. et al., 2020). Developmental processes contributing to the establishment of shoot architecture, such as tillering, vegetative vigour, and dwarfing, are crucial agronomic traits affecting crop yield and can be manipulated by the application of SLs. For rice and wheat, the proper number of tillers is one of the significant factors that improves the grain yield, which may be related to SL exudation (Song et al., 2017; Zhao et al., 2019). In the case of *Brassica napus*, biomass increased the following spray with GR24 in growth chambers (Ma et al., 2017). Stem thickness was reduced in the SL signalling-deficient mutants of *Arabidopsis* and pea (Agusti et al., 2011). The specific features of SL action in the stem and root thickening can be exploited to reduce lodging susceptibility in cereal crops and increase timber production in silviculture. Newer opportunities for SL applications are likely to arise based on the studied examples described earlier.

The root system architecture plays a key role in optimising nutrient use efficiency and water acquisition, thereby enabling plant growth in nutrient-poorer soils. SLs regulate plant root development, although the specific effects vary across species and growth conditions. SL biosynthesis-deficient mutant in *Arabidopsis* developed more lateral roots (LRs) under optimal growth conditions, whereas an opposite effect was observed under the P-deficient conditions (Ruyter-Spira et al., 2011). ET blocks auxin-driven LR formation (Lewis et al., 2011). SLs translate P starvation signals into growth cues in the roots and interact with ET and auxin to exert their impact. In *Arabidopsis* and *pea*, SL signalling-deficient mutant exhibited more ARs, indicating that SLs suppress AR number (Rasmussen et al., 2012). Indeed, GR24 restored AR formation in the SL biosynthesis-deficient mutant *D10* but not in the SL signalling-deficient mutant *D3* of rice (Sun H. et al., 2015). The exact mechanism underlying the involvement of SLs in root development remains unclear because of conflicting data for different species. The highly complex hormonal interactions between SLs and other classes of phytohormones may all contribute to eventual root architectural modifications. Thus, plants can benefit from these interactions during development and adaptation to a changing environment (refer to the “Crosstalk between strigolactones and other hormones in plant growth and development, and in response to environmental changes” section for details).

### Improvement of Nutrient Acquisition

Nutrient availability, particularly P deficiency, in agricultural soils affects SL exudation and distribution (Yoneyama et al., 2012). The increase in SL content is consistent with the expression of SL biosynthesis genes in rice roots, and this expression is elevated under N- or P-limiting conditions compared with that under controlled normal development conditions (Sun et al., 2014). Additionally, the ABC transporter Pleiotropic Drug Resistance1 (PDR1) translocated synthesised SLs from the root to shoot, and its transcription level increased in the roots of N- or P-deficient petunia and *Lotus japonicus* (Kretzschmar et al., 2012; Liu et al., 2015; Shiratake et al., 2019). The key genes, triad *IPS1*-miR399-*PHO2* and the high-affinity P transporter *LePT2*, were involved in the response of tomato plants to low P availability (Gamir et al., 2020). No matter growing with P or not, SL biosynthesis-deficient tomato mutant could not efficiently activate most mechanisms associated with the P starvation response compared with wild-type plants (Santoro et al., 2021). SLs also act as molecular cues favouring arbuscular mycorrhizal (AM) symbiosis establishment in the rhizosphere, particularly increasing their access to nourishment and moisture from the nutrient-limited soil (Kapulnik and Koltai, 2014; Sun J. et al., 2015; Waters et al., 2017).

Strigolactones facilitate plants in responding to N and P starvation by shaping the above-and belowground architecture. Shoot growth and tiller production in rice were inhibited under the suboptimal P concentration, whereas SL signalling-deficient mutant (*D3*) and SL biosynthesis-deficient mutant (*D10*) showed no adverse effects (Luo et al., 2018). SLs positively regulated in stimulating PR length in rice, wheat, and tomato under limited P resources (Jamil et al., 2011; Yoneyama et al., 2012; Santoro et al., 2020). A similar effect was observed for root elongation in rice (Arite et al., 2012). SLs are vital for nitric oxide-regulated rice seminal root elongation during P and N starvation (Sun et al., 2016). The seminal root length of SL biosynthesis-deficient mutant (*D10* and *D27*) and SL signalling-deficient mutant (*D3*) in rice decreased under low-P conditions. By contrast, all these SL-related mutants presented increased LR density during P starvation compared with wild-type plants (Sun et al., 2014). This negative effect on LR growth is attributable to the SL-mediated inhibition of polar auxin transport from shoots to roots and alteration of auxin distribution in roots (Sun et al., 2014). The potential of SLs in nutrient starvation response is valuable in developing strategies to improve nutrient use efficiency and productivity in low-fertility soils.

### Mediation of Plant Tolerance to Drought and Salinity

Climatic changes increase drought and soil salinity, reducing crop yield in the affected areas. SLs participate directly in plant tolerance to abiotic stresses. During the analysis of the promoter sequences of SL biosynthesis genes in *Arabidopsis*, *cis*-acting sequences that specifically bind to drought and salt-responsive transcription factors were identified (Marzec and Muszynska, 2015). Under drought conditions, the SL analogue AB01 improved the grain yield and kernel weight of maize and sunflower (Chesterfield et al., 2020). SL biosynthesis- or signalling-deficient mutants are hypersensitive to unfavourable environmental conditions such as drought, salt, and osmotic stress (Zhang et al., 2018; Qiao et al., 2020; Zheng et al., 2021). The expression levels of SL biosynthesis genes (*SlCCD7* and *SlCCD8*) and SL content decreased in tomato roots under drought stress (Visentin et al., 2016). By contrast, SL levels were elevated in rice roots in response to water withholding-induced dehydration (Haider et al., 2018). Monocots and dicots may adopt different survival strategies to cope with the water deficit.

The abundance of AM fungi in the rhizosphere of lettuce plants increased in response to salinity-induced SL secretion from roots (Aroca et al., 2013). However, SL biosynthesis-deficient mutant of rice exhibited lower AM colonisation than wild-type plants (Kobae et al., 2018). Drought stress-induced SL production in lettuce and tomato further triggered the growth of AM fungi, thus improving drought resistance (Ruiz-Lozano et al., 2016). SLs promote communication between the host and beneficial soil microorganisms, an eco-friendly strategy, and allow plants to better withstand environmental changes.

## Crosstalk Between Strigolactones and Other Hormones in Plant Growth and Development, and in Response to Environmental Changes

### Strigolactones and Abscisic Acid

The correlation between ABA and SLs is critical for regulating multiple physiological mechanisms and adaptation to environmental changes in plants. The ABA importer genes *ABCG22/AT5G06530* and *ABCG40/AT1G15520* were downregulated in the SL signalling-deficient mutant *max2* of *Arabidopsis* under well-watered and dehydrated conditions (Ha et al., 2014; Ruiz-Lozano et al., 2016). SLs induce tolerance to drought and salt stress largely by activating ABA signalling. Resistance to drought associated with slower stomatal closure, which was attributed to ABA insensitivity, was impaired in the SL biosynthesis-deficient mutant of *L. japonicus* (Liu et al., 2015). Similar ABA-SL crosstalk was demonstrated in which GR24 pre-treatment alleviated the adverse effects of salt stress in rice and grapevine seedlings and better induced stomatal closure (Min et al., 2019; Ling et al., 2020). The effect of SLs on stomatal closure depends on ABA synthesis, transport, and sensitivity (Visentin et al., 2020). Another recent study found that the SL biosynthesis-deficient mutant *D10* and *D17* and the SL signalling-deficient mutant *D13* of rice had higher ABA accumulation than wild-type plants, resulting in induced drought tolerance (Haider et al., 2018). By contrast, the low-ABA-producing line D27 was susceptible to drought implying that D27 participates in the ABA signalling pathway (Haider et al., 2018). However, the mechanism by which D27 links ABA to SL has not been elucidated. Apart from their role in drought resistance, the positive role of SLs in the cold and heat stress response is associated with ABA biosynthesis. GR24^5DS^ application enhanced heat and cold tolerance in tomato, whereas the ABA-deficient mutant compromised the GR24^5DS^ effects, implying that SL, at least partially in an ABA-dependent manner, allows plants to flexibly acclimate to and overcome these stress conditions (Chi et al., 2021).

Strigolactones and ABA both participate in the regulation of branching or tillering, and ABA acts as downstream of SLs and BRC1 in *Arabidopsis* (González-Grandío et al., 2017; Wang B. et al., 2018; Wang and Bouwmeester, 2018; Wang et al., 2020). SLs mediating axillary bud outgrowth are involved in degrading SMXL6 and releasing BRC1 transcriptional repression, thereby inducing *HB40*/*OsHOX12* expression, activating *AtNCED3*/*OsNCED1* expression, and promoting ABA accumulation in the lateral buds of *Arabidopsis* or shoot bases of rice (Liu et al., 2020; Wang et al., 2020). Indeed, ABA supply inhibits tiller bud growth and suppresses the formation of unproductive upper tillers in rice, but its contribution is less than that of SLs (Liu et al., 2020).

During abiotic stress, ABA levels increase rapidly, but SL content may vary in different species. ABA positively regulated SL levels and the expression of signalling genes to improve salt stress acclimatisation and resistance in *Sesbania cannabina* (Ren et al., 2018). Similarly, plant resilience to water deprivation is promoted through the upregulation of the transcript levels of SL biosynthesis genes in rice root extracts (Haider et al., 2018). However, both the SL level and the SL gene expression in tomato and *L. japonicus* decreased under osmotic stress (Liu et al., 2015; Visentin et al., 2016). Breeding for potentially drought-tolerant crop varieties by SL signal upregulation requires further exploration.

### Strigolactones and Cytokinin

As physiological processes vary, so do the interactions between SLs and CK. CK and SLs regulate separate processes and function independently in adventitious rooting, synergistically controlling LR development, but antagonistically regulating axillary bud outgrowth (Dun et al., 2013; Hu et al., 2014; Manandhar et al., 2018; Faizan et al., 2020).

Strigolactones and CK interact directly in buds, and they integratively promote the transcriptional regulation of *BRC1* in *Arabidopsis* and pea or *FINE CULM 1*, an orthologous gene of *BRC1*, in rice (Braun et al., 2012; Dun et al., 2013; Xu et al., 2015). BRC1 is known to modulate the bud activation potential in several species by acting as an important hub of regulatory signals controlling bud outgrowth (Martin-Trillo et al., 2011; Nicolas et al., 2015; Shen et al., 2019). The antagonistic action of CK and SL mediates the inhibitory effect of auxin on bud outgrowth (Rameau et al., 2015; Barbier et al., 2019). In rice, SLs activate CK catabolism to alter the shoot architecture *via* cytokinin oxidase/dehydrogenase 9 (OsCKX9) activity (Duan et al., 2019). Therefore, along with induced activation of OsCKX9, SLs may affect the CK content through crosstalk with auxin. In addition, high sugar levels were found to inhibit SL perception, notably by directly targetting SL signalling (Dierck et al., 2016; Bertheloot et al., 2020; Patil et al., 2021). Sugars were also found to upregulate the levels of CK, which acts antagonistically with SLs (Barbier et al., 2015; Kiba et al., 2019; Salam et al., 2021). However, the exact role of CK in the sugar response remains undetermined. During branching, HEXOKINASE1 mediates the sugar signalling pathway, allowing plants to fine-tune the shoot architecture, and interacts with CK and SLs (Barbier et al., 2021).

GR24 inhibits PR elongation by altering *PIN* gene transcription, which is mediated by Short Hypocotyl2 (SHY2) through CK signalling components (Jiang et al., 2016). The CK response transcription factor (ARR1) directly binds to specific promoter sequences of the protein SHY2 and activates its expression, which in turn represses the *PIN* genes, while auxin stalls LR formation by SHY2-mediated repression of PIN activity (Sengupta and Reddy, 2018). SHY2 acts as a node-linking hormone that regulates root meristem development. SLs may affect the endogenous levels and distribution of each hormone, coordinately controlling the root (meristem) size.

Cytokinins and SLs play opposite regulatory roles in plant adaptation to drought. In CK-depleted and CK-signalling mutants of *Arabidopsis*, CKs and CK-signalling components were found to negatively regulate plant drought acclimation (Nishiyama et al., 2013; Nguyen et al., 2016). Conversely, SLs positively regulate drought resistance-related physiological traits by altering stomatal density and stomatal conductance (Ha et al., 2014; Zhang et al., 2018). In addition, downregulation of CK catabolism genes (*CKX1*, *CKX2*, *CKX3*, and *CKX5*) following dehydration was observed in the SL signalling-deficient mutant *MAX2* compared with wild-type plants (Ha et al., 2014). This indicates that the SL signal might have an antagonistic effect on the CK content, which can be confirmed by detailed studies on SL biosynthesis and signalling mutants under drought stress because MAX2 appears to be shared by both SL signalling and karrikin signalling pathways (Soundappan et al., 2015).

### Strigolactones and Auxin

Strigolactones and auxin synergistically regulate shoot branching and root development (Crawford et al., 2010; Ma et al., 2020; Zhang J. et al., 2020). SL-mediated regulation of shoot branching is tightly linked to PIN-dependent auxin transport, specifically its canalisation (Crawford et al., 2010; Shinohara et al., 2013). This is supported by the fact that SL biosynthesis-deficient mutants *MAX4-5* and *D27-1* exhibit enhanced accumulation of the PIN1 auxin efflux carrier on the basal plasma membrane (Bennett et al., 2016) and that GR24 treatment can induce PIN1 endocytosis and reduce auxin transport during SL biosynthesis in *Arabidopsis* but not in response mutants (Shinohara et al., 2013). SLs inhibit auxin feedback on PIN polarity and clathrin-mediated endocytosis of PIN proteins through D14- and MAX2-mediated signalling pathways (Zhang J. et al., 2020). However, exogenous SLs can still suppress bud outgrowth in auxin transport inhibitor 1-N57 naphthylphthalamic acid-treated shoots, suggesting the existence of another mechanism of bud growth inhibition by SLs. This also suggests that SL acts directly on bud outgrowth independent of polar auxin transport (Chabikwa et al., 2019). Many questions about the exact mechanism of SL action and perception may be answered by examining some promising candidates as downstream mediators of SL signalling (Brewer et al., 2015).

During root development, SLs regulate LR and RH development by changing auxin distribution (Haq et al., 2017; Sun et al., 2019). Polar auxin transport mainly depends on the auxin efflux protein PINs. This protein creates local auxin maxima to form the basis for root initiation and elongation (Zhang Y. et al., 2020). GR24 reduced IAA distribution and modulated AR formation by downregulating the levels of PIN family genes in rice (Sun H. et al., 2015). However, in the presence of exogenous auxin, the PIN gene expression level in the PR tip of *Arabidopsis* was not affected by GR24 treatment (Ruyter-Spira et al., 2011). A similar crosstalk between SLs and auxin occurs in the regulation of RH development where SL-mediated reduction of auxin accumulation within root cells results in high RH length and density (Koltai et al., 2010). These root responses are typical to P-deficient conditions (Santoro et al., 2020). The auxin-responsive element of the bHLH transcription factor ROOT HAIR DEFECTIVE SIX-LIKE 4 positively regulates genes involved in cell processes key to RH growth under the P-deficient condition (Bhosale et al., 2018; Zhu et al., 2020). These backgrounds clarify that RSL4 may function as a common integrator for the crosstalk between SLs and auxin in modulating RH elongation (Marzol et al., 2017). RH morphogenesis is driven by interacting processes controlled by complex hormone signalling. How these signalling components induce SL biosynthesis and signalling according to the P status at the molecular level remains unclear. Further studies should focus on cloning genes involved in RH mutants and undertaking reverse genetics and mutant complementation experiments to gain extended knowledge on signalling networks.

### Strigolactones and Ethylene

**FIGURE 3:**
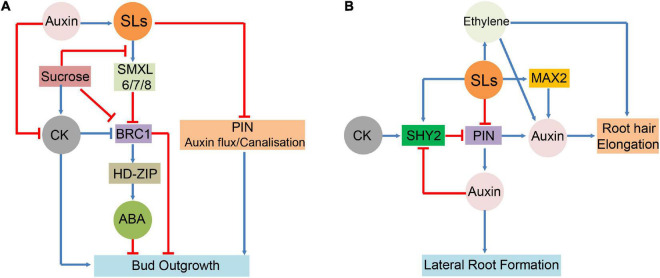
Interactions between SLs and hormones in major growth and developmental process. **(A)**: SLs functions and interactions with ABA, auxin, and CK in the regulation of bud outgrowth. **(B)**: Involvement of SLs in the hormonal control of lateral root and root hair (RH). 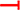 signify inhibitory effect; 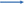 signify stimulatory effect.

Strigolactones have also been demonstrated to interact with ET signalling and control RH elongation. ET signalling-deficient *ein2* and *etr1* mutants exhibited no influence of SLs on RH morphogenesis (Kapulnik et al., 2011). In *Arabidopsis*, RH elongation was enhanced by GR24 treatment alone but not by treatment with the ET biosynthesis inhibitor aminoethoxyvinylglycine, even in the presence of GR24 (Lee and Yoon, 2020). This indicates that ET is necessary for promoting SL-mediated RH elongation. SLs adjust the balance between auxin and ET signalling pathways to activate different developmental programmes in response to soil nutrient limitations, thereby controlling their own biosynthesis in roots under these conditions. Under P-sufficient conditions, SLs interact with ET and promote auxin signalling transduction (Koltai, 2013). ET forms a crosstalk junction between SLs and auxin pathways in modulating RH formation. Together, these hormones probably create a deliberately coordinated network for regulating plant growth and its response to adverse growth conditions (Figure 3).

### Strigolactones Application Challenges and Future Directions

With the recent discovery of a hormonal function for SLs, SL-mediated regulation of plant development has been explored extensively. Phenotypic plasticity is crucial for plants adapting to changing or extreme abiotic environments. Modification of SL signalling pathways to create an optimal crop architecture is a pivotal physiological strategy in improving nutrient uptake and utilisation, crop productivity, and resilience. As novel molecular technologies have increased the feasibility of genetic improvement of crops, variants with modified SL profiles, for example, transgenic rice (OsMADS57 and OsTB1), have led to increased grain yield with upregulated SL response pathways modulating tillering. Furthermore, ontogenetic modification of the SL transport signal, such as overexpression of the SL transporter PDR1, might be useful for obtaining a potential breeding stock. Applying SL analogues for shaping the plant architecture, improving their performance and resistance, and enhancing AM colonisation are of high potential value. Cheaper sources of SLs analogues are required for large-scale agricultural applications.

Similar to other phytohormones, SL biosynthesis and activity are regulated by multiple levels of crosstalk in hormonal networks under suboptimal environmental conditions (Table 1). As the interface between the plant and soil, roots are more exposed to adverse soil conditions than the aerial parts of the plant. The roots’ perception of the environment influences plant morphology. Progress has been made in understanding how different phytohormones facilitate root growth plasticity. More components involved in these processes and spatial temporal relationships between these components need to be identified. Additional experimental and theoretical studies are warranted to carefully understand the different contributions of SLs and these hormones to the whole plant level of organisation. The endogenous levels of phytohormones need to be optimised to maximise stress-responsive crosstalk between multiple hormones.

**TABLE 1:** Effects of strigolactones and hormones crosstalk on various plant species.

Plant hormones	Investigated Species	Type of experiment	SL effect	Antagonism or synergism	References
ABA	*Arabidopsis thaliana*	SLs-response max2 mutant	Effect ABA import	Synergism	Ha et al., 2014; Ruiz-Lozano et al., 2016
	*L. japonicus*	SL-biosynthesis mutant	Slow stomatal closure	Synergism	Liu et al., 2015
	Rice and grapevine seedlings	synthetic GR24	Induces stomatal closure	Synergism	Min et al., 2019; Ling et al., 2020
	Rice	SL-deficient mutants D10 and D17 SL-perception mutant D13	Induce drought tolerance	Synergism	Haider et al., 2018
CK	*Arabidopsis thaliana*	GR24	Inhibits the elongation of the primary root	Synergism	Jiang et al., 2016
	Rice	SLs-insensitive tiller dwarfing mutants	Increase auxin level	Synergism	Sun et al., 2019
IAA	Rice	GR24	Reduced IAA distribution and modulated AR formation	Antagonis	Sun H. et al., 2015
ET	*Arabidopsis thaliana*	ET signalling deficient *ein2* and *etr1* mutants	Eliminate the influence of SLs on the Root morphogenesis	Synergism	Kapulnik et al., 2011

## Author Contributions

FW, YG, and WY wrote the manuscript. NS and JZ edited the manuscript. All the authors discussed and created the review’s outline.

## Conflict of Interest

The authors declare that the research was conducted in the absence of any commercial or financial relationships that could be construed as a potential conflict of interest.

## Publisher’s Note

All claims expressed in this article are solely those of the authors and do not necessarily represent those of their affiliated organizations, or those of the publisher, the editors and the reviewers. Any product that may be evaluated in this article, or claim that may be made by its manufacturer, is not guaranteed or endorsed by the publisher.
